# The Effect of Salvianolic Acid A on Tumor-Associated Macrophage Polarization and Its Mechanisms in the Tumor Microenvironment of Triple-Negative Breast Cancer

**DOI:** 10.3390/molecules29071469

**Published:** 2024-03-26

**Authors:** Chao Tang, Shi-Ting Jiang, Cheng-Xia Li, Xiao-Fang Jia, Wen-Li Yang

**Affiliations:** 1 Institute for Cancer Medicine, School of Basic Medical Sciences, Southwest Medical University, Luzhou 646000, China; tc920803@163.com (C.T.); jiangshiting2023@163.com (S.-T.J.); lcx2512987955@163.com (C.-X.L.); jxf0416@163.com (X.-F.J.); 2 Department of Biochemistry and Molecular Biology, School of Basic Medicine, Southwest Medical University, Luzhou 646000, China

**Keywords:** salvianolic acid A, tumor-associated macrophage, triple-negative breast cancer, macrophage polarization

## Abstract

Triple-negative breast cancer (TNBC) is the most aggressive subtype of breast cancer, with a high degree of malignancy and poor prognosis. Tumor-associated macrophages (TAMs) have been identified as significant contributors to the growth and metastasis of TNBC through the secretion of various growth factors and chemokines. Salvianolic acid A (SAA) has been shown to have anti-cancer activities. However, the potential activity of SAA on re-polarized TAMs remains unclear. As there is a correlation between the TAMs and TNBC, this study investigates the effect of SAA on TAMs in the TNBC microenvironment. For that purpose, M2 TAM polarization was induced by two kinds of TNBC-conditioned medium (TNBC-TCM) in the absence or presence of SAA. The gene and protein expression of TAM markers were analyzed by qPCR, FCM, IF, ELISA, and Western blot. The protein expression levels of ERK and p-ERK in M2-like TAMs were analyzed by Western blot. The migration and invasion properties of M2-like TAMs were analyzed by Transwell assays. Here, we demonstrated that SAA increased the expression levels of CD86, IL-1β, and iNOS in M2-like TAMs and, conversely, decreased the expression levels of Arg-1 and CD206. Moreover, SAA inhibited the migration and invasion properties of M2-like TAMs effectively and decreased the protein expression of TGF-β1 and p-ERK in a concentration-dependent manner, as well as TGF-β1 gene expression and secretion. Our current findings for the first time demonstrated that SAA inhibits macrophage polarization to M2-like TAMs by inhibiting the ERK pathway and promotes M2-like TAM re-polarization to the M1 TAMs, which may exert its anti-tumor effect by regulating M1/M2 TAM polarization. These findings highlight SAA as a potential regulator of M2 TAMs and the possibility of utilizing SAA to reprogram M2 TAMs offers promising insights for the clinical management of TNBC.

## 1. Introduction

Breast cancer (BC) is currently the most common type of cancer among women worldwide and the leading cause of cancer deaths in women [1,2]. Triple-negative breast cancer (TNBC), an aggressive subtype, accounts for approximately 15–20% of breast cancers and remains the most difficult subtype to cure [3]. Currently, chemotherapy is the main treatment for TNBC but chemotherapy is prone to drug resistance and strong adverse effects, which is an urgent problem that needs to be addressed. There is still a need to investigate alternative strategies using drugs that have the potential to improve clinical benefits with less toxicity. The development of new treatment options and targets for TNBC is, thus, an urgent necessity.

Tumor-associated macrophages (TAMs) represent a large portion of the tumor microenvironment (TME) and have been proven to be involved in multiple biological processes in TNBC [4]. In TME, macrophages (Mφ) can polarize into M1 and M2 TAMs, each demonstrating distinct physiological functions. M1 TAMs exhibit anti-tumor properties that promote immune responses and inhibit tumor growth. On the other hand, M2 TAMs possess pro-tumor characteristics that support angiogenesis, tissue remodeling, and immunosuppression [5]. The major subtype of TAMs is M2 TAMs, which can be induced via Th2 cytokines, such as IL-4, IL-13, and M-CSF. Cytokines expressed by TAMs are closely related to the phenotype of TAMs and play an important role in promoting breast cancer growth, metastasis, and extracellular matrix remodeling [6], which is valuable for learning the phenotypic transformation and the reprogramming of M2 TAMs [7]. Therefore, a more complete understanding of the TAMs and their regulators is essential to developing immune-based therapies targeted towards TNBC.

Salvianolic acid A (SAA), a natural compound obtained from the Chinese herb Danshen [8], has demonstrated promising anti-tumor properties in various types of tumors [9]. However, the effect of SAA on TAM polarization in TNBC remains unclear. The aim of this study was to investigate the effect of SAA on the polarization of TAMs induced by TNBC-conditioned medium (TNBC-TCM) and its potential mechanisms.

## 2. Results

### 2.1. TNBC Cell-Conditioned Medium Induced the Polarization of M2-like TAMs

**Figure 1:**
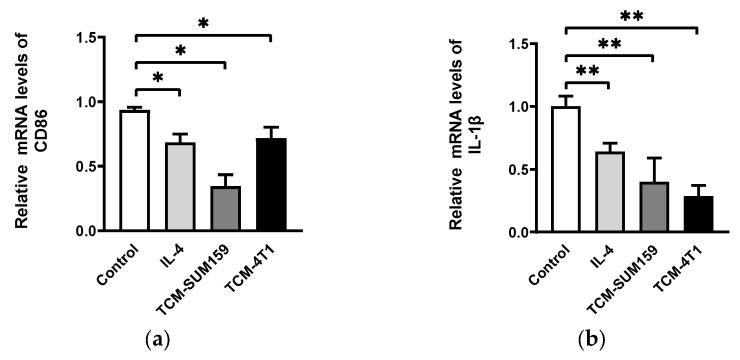

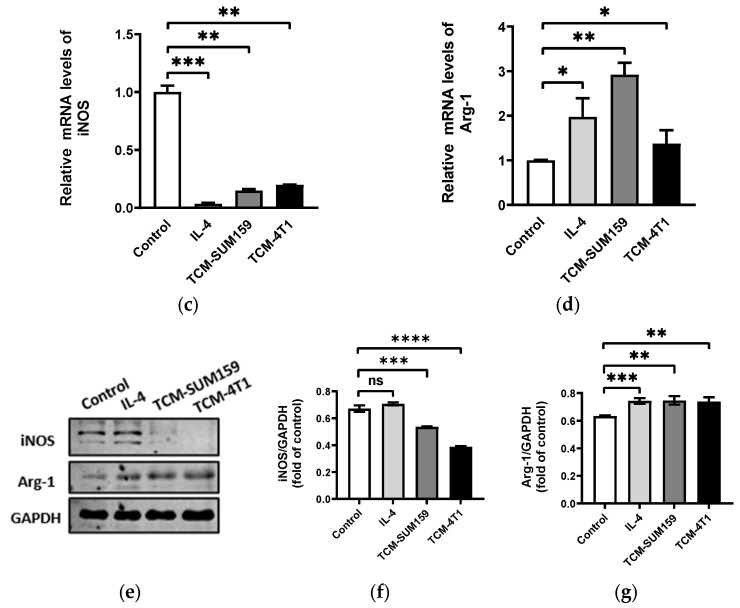
The TNBC-cell-conditioned medium induced the polarization of M2-like TAMs. After treating RAW264.7 cells with IL-4, TCM-SUM159, and TCM-4T1, the relative mRNA expression levels of (**a**) CD86, (**b**) IL-1β, (**c**) iNOS, and (**d**) Arg-1 were measured via qRT-PCR. (**e**–**g**) The protein levels of iNOS and Arg-1 were evaluated through Western blot analysis. Data are presented as the mean ± SD (*n* = 3). * *p* < 0.05; ** *p* < 0.01; *** *p* < 0.001; **** *p* < 0.0001.

To mimic the TME of breast cancer, the RAW264.7 cells were first exposed to the conditional medium of the TNBC cells (SUM159 and 4T1) to induce TAM polarization. M1 TAMs promote immune-mediated anti-tumor activity via the production of CD86 and inducible nitric oxide synthase (iNOS), which contributes to the Th1 response in the tumor microenvironment [10]. The cytokine interleukin-1β (IL-1β) is a key mediator of the inflammatory response and also one of the markers of M1 TAMs [11,12]. Arginase 1 (Arg-1) is one of the key factors in the suppressive function of TAMs and its expression is widely detected in immature and functional M2 TAMs [10,13]. Therefore, we chose CD86, iNOS, and IL-1β to identify M1 TAMs and Arg-1 to identify M2 TAMs. Quantitative real-time PCR analysis revealed that IL-4, TCM-SUM159, and TCM-4T1 downregulated the relative mRNA expression of CD86 (Figure 1a), IL-1β (Figure 1b), and iNOS (Figure 1c) while upregulating the relative mRNA expression of Arg-1 (Figure 1d) compared to the untreated macrophages. The results of Western blot analysis showed that the protein expression of iNOS was decreased in the TCM-SUM159 and TCM-4T1 groups and Arg-1 was increased in the IL-4, TCM-SUM159, and TCM-4T1 groups (Figure 1c–e). Together, these results demonstrate that macrophages are polarized into M2-like TAMs by the TNBC cell-conditioned medium (TCM-SUM159 and TCM-4T1).

### 2.2. TGF-β1 Expression Was Upregulated and the ERK Signalling Pathway Was Activated in Macrophages Co-Cultured with TCM-TNBC

**Figure 2:**
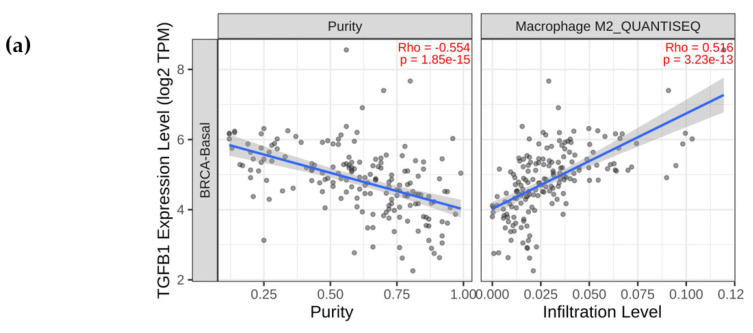

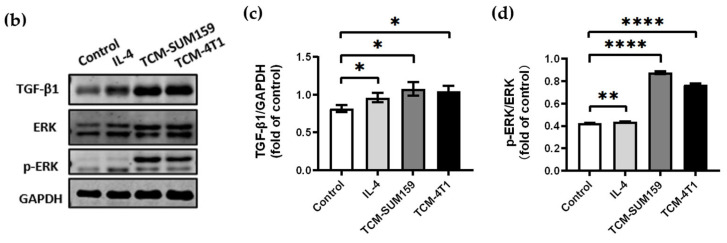
ERK pathway was activated in M2-like TAMs induced via the TNBC-cell-conditioned medium. (**a**) Correlation between expression of TGF-β1 and M2 macrophage infiltration in breast cancer using TIMER2.0 database. (**b**–**d**) After treating M2-like TAMs with the media (TCM-SUM159 and TCM-4T1), the protein expression levels of TGF-β1, ERK, and p-ERK were evaluated using Western blot analysis. Data are presented as the mean ± SD (*n* = 3). * *p* < 0.05; ** *p* < 0.01; **** *p* < 0.0001.

M2 TAMs can express high levels of TGF-β1, which can selectively activate M2 TAMs [14], thus creating a vicious cycle that continuously stimulates the production of M2 TAMs. Using the Timber 2.0 database, we found a positive correlation between TGF-β1 expression and M2 macrophage infiltration in human breast cancer cells (r = 0.374, *p* < 0.001, Figure 2a). After macrophages were treated with IL-4, TCM-SUM159, and TCM-4T1, Western blot analysis showed that the protein expression level of p-ERK and TGF-β1 were upregulated, while total ERK remained unaffected, compared to the untreated macrophages (Figure 2b–d). These results indicate that in macrophages co-cultured with TCM-TNBC, TGF-β1 expression was upregulated and the ERK signaling pathway was activated.

### 2.3. SAA Mediated the mRNA Expression of Cytokines in M2-like TAMs Induced by the TNBC-Cell-Conditioned Medium

**Figure 3:**
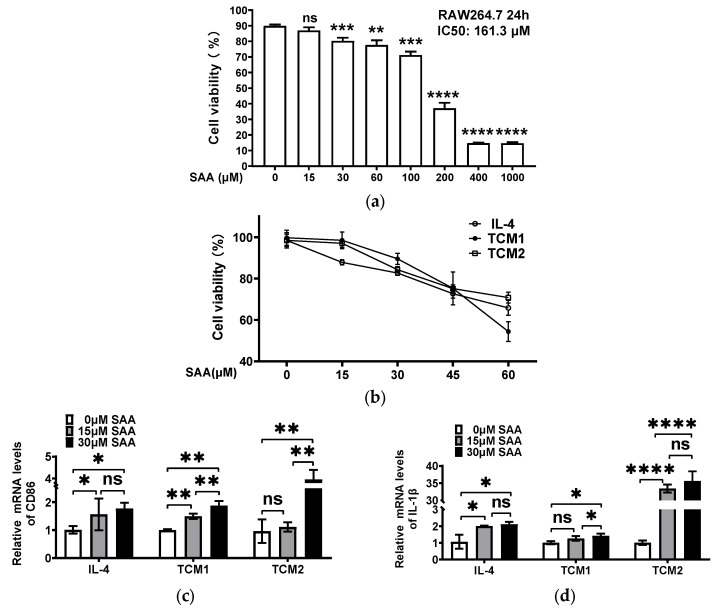

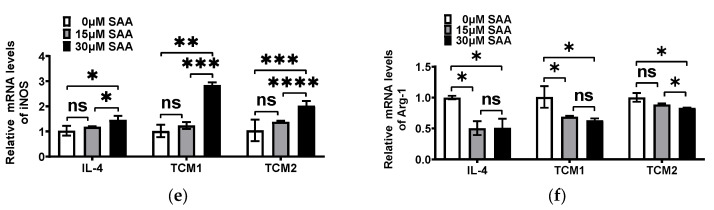
SAA mediated the mRNA expression levels of cytokines in M2-like TAMs induced by the TNBC-cell-conditioned medium. (**a**) RAW264.7 cells were treated with increasing concentrations (0–1000 μM) of SAA for 24 h and the IC50 values were assessed via a CCK-8 assay. ** *p* < 0.01, *** *p* < 0.001, **** *p* < 0.0001 versus control. (**b**) TCM-SUM159—TCM1 and TCM-4T1—TCM2. RAW264.7 cells were exposed to IL-4, TCM1, and TCM2 in the presence or absence of SAA (0–60 μM); the cell viability of the M2-like TAMs was detected via a CCK-8 assay. Following induction with IL-4, TCM1, and TCM2, RAW264.7 cells were simultaneously treated with SAA and the relative mRNA expression levels of (**c**) CD86, (**d**) IL-1β, (**e**) iNOS, and (**f**) Arg-1 were measured via qRT-PCR. * *p* < 0.05; ** *p* < 0.01; *** *p* < 0.001; **** *p* < 0.0001.

Although SAA can inhibit breast cancer development and progression [9], it remains unclear whether SAA can regulate the polarization and malignant behavior of TAMs in the tumor microenvironment. In order to investigate the effect of SAA on M2-like TAMs, we determined the IC50 value of SAA on RAW264.7 cells, which was found to be 161.3 μM (Figure 3a). 

We selected a concentration range of 0–60 μM of SAA to determine the appropriate concentration for M2-like TAMs. SAA (0, 15, 30, 45, and 60 μΜ) were added to macrophages co-cultured with IL-4, TCM-SUM159, and TCM-4T1 for the CCK-8 assay. The results indicated that cell viability was less than 80% at SAA concentrations of 45 and 60 μM (Figure 3b). Similarly, the post-dosing group showed the same outcomes (Supplementary Figure S1a). These findings demonstrated that high concentrations of SAA exhibit significant cytotoxic effects on M2-like TAMs induced by the TCM-TNBC, whereas SAA concentrations of 15 and 30 μM showed no significant cytotoxic effects. Consequently, SAA concentrations of 15 and 30 μM were used in subsequent experiments.

To assess whether SAA could influence the RAW264.7 cells, RAW264.7 cells were treated with 15 and 30 μM of SAA for 24 h to detect the mRNA expression of TAM markers (as the negative control). The results showed that 15 and 30 μM SAA had no effect on RAW264.7 cells (Supplementary Figure S1b). Afterward, we polarized the RAW264.7 cells with IL-4, TCM-SUM159 (TCM1), and TCM-4T1 (TCM2) in the presence or absence of SAA (15 and 30 μM) to analyze the effect of SAA on M2-like TAMs. The results showed that, in the simultaneous dosing group, after treatment with SAA, the mRNA levels of IL-1β (Figure 3c), CD86 (Figure 3d), and iNOS (Figure 3e) were increased and the mRNA levels of Arg-1 (Figure 3f) were decreased in a concentration-dependent manner. The same results were obtained in the post-dosing group (Supplementary Figure S1c–f). Together, these results indicate that 15 and 30 μM SAA may inhibit the progression of RAW264.7 cells towards M2-like TAMs.

### 2.4. SAA Inhibited the Progression of RAW264.7 Cells Polarization towards M2-like TAMs

**Figure 4:**
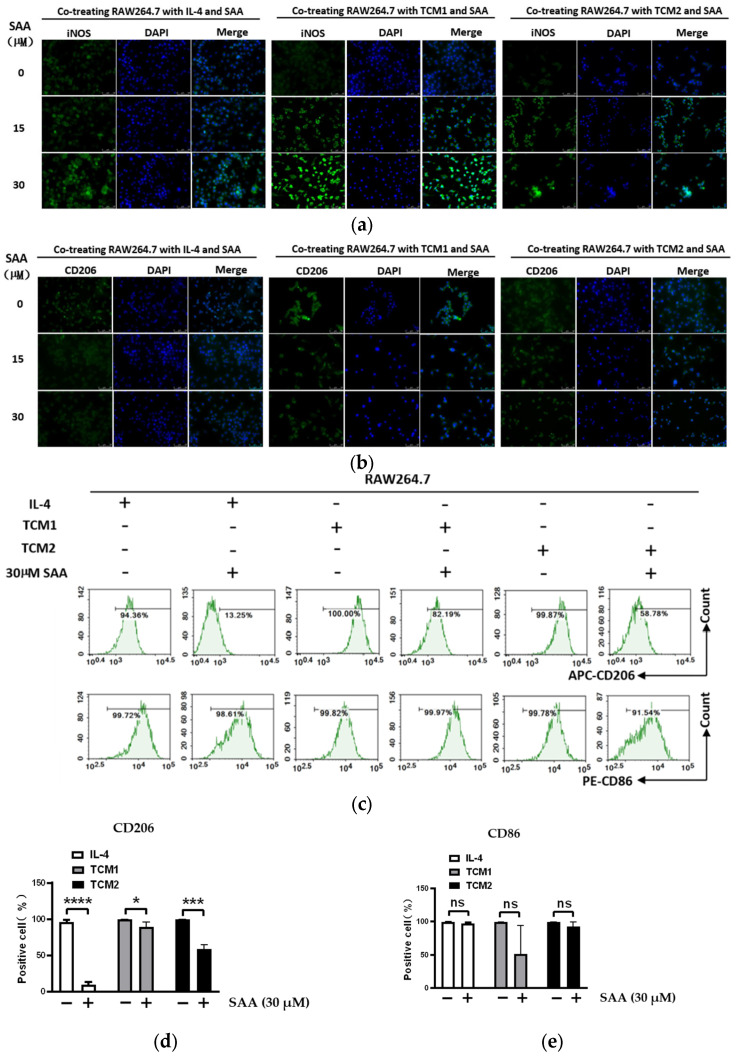
SAA inhibited the progression of RAW264.7 cell polarization towards M2-like TAMs. Immunofluorescence staining was performed to detect the expression levels of (**a**) iNOS and (**b**) CD206, as shown in green, while the nuclei appear in blue (400×). (**c**–**e**) Flow cytometric analysis of surface markers of CD86 and CD206 in M2-like TAMs. * *p* < 0.05; *** *p* < 0.001; **** *p* < 0.0001.

CD206 is widely accepted as the canonical marker for M2a TAMs [15]; we have used cellular immunofluorescence (IF) to detect the expression of iNOS and CD206. The results showed that after treatment with SAA (15 and 30 μM) in the simultaneous dosing and post-dosing groups, the protein expression level of iNOS was increased (Figure 4a, Supplementary Figure S2a) and the protein expression levels of CD206 was decreased (Figure 4b, Supplementary Figure S2b). 

In recent years, much evidence has shown that M1 TAMs express abundant CD86, whereas M2 TAMs express high levels of CD206. It has been demonstrated that the low presence of CD86 M1 TAMs and the high presence of CD206 M2 TAMs correlate significantly with an aggressive tumor phenotype and a poorer prognosis [16,17]. Therefore, flow cytometry (FCM) was used to detect CD86 and CD206 for further identification of M2 TAMs. The results showed that after treatment with SAA (15 and 30 μM), the percentage of CD206+ M2-like TAMs was decreased (Figure 4c,d) but CD86+ TAMs exhibited no change (Figure 4c,d). Similar results were observed in the post-dosing group (Supplementary Figure S2c,d). 

Considering the different roles of CD86 in different tumors, for example, infiltration of CD86 TAMs indicates a good prognosis in colorectal cancer [18], whereas in patients with multiple myeloma, CD86 TAMs do not correlate with tumor progression [19]. In contrast, CD206 is the most commonly used M2 TAM marker and has been directly associated with poor prognosis in a wide range of tumors [19,20,21]. Taken together, these results demonstrate that SAA inhibits the progression of RAW264.7 cell polarization towards M2-like TAMs.

### 2.5. SAA Inhibited the Migration and Invasion of M2-like TAMs Induced by the TCM-TNBC

**Figure 5:**
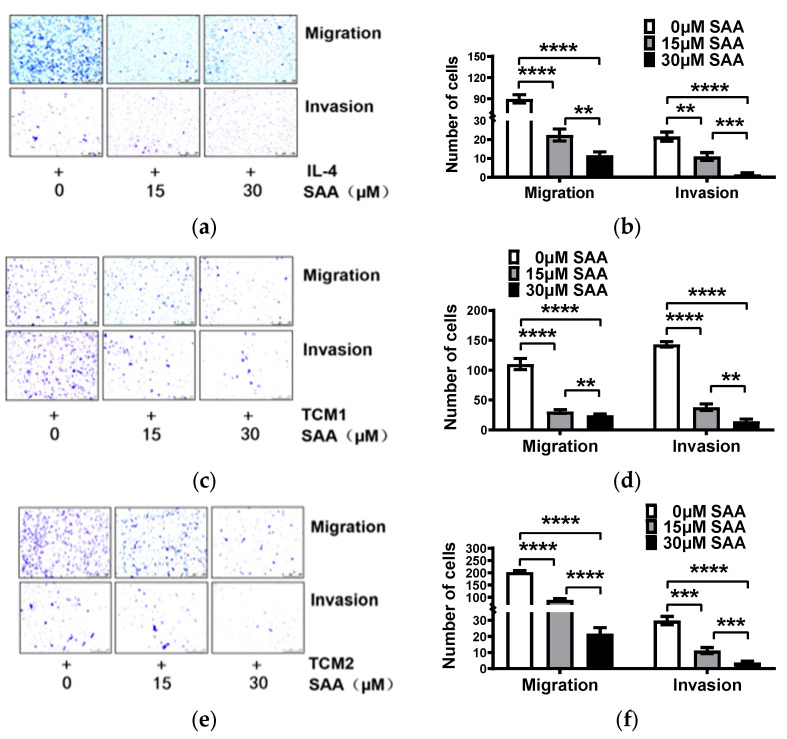
SAA inhibited the migration and invasion of M2-like TAMs induced by the TCM-TNBC. (**a**,**b**) IL-4, (**c**,**d**) TCM1, and (**e**,**f**) TCM2 induced M2-like TAMs after treatment with 15 or 30 μM SAA concentrations in migration and invasion assays. ** *p* < 0.01; *** *p* < 0.001; **** *p* < 0.0001.

In the above-mentioned results, SAA successfully inhibited the progression of RAW264.7 cells towards M2-like TAMs. Next, we measured the modulatory effects of SAA on the invasion and migration properties of M2-like TAMs. Remarkably, the results suggest that SAA (15 and 30 μM) inhibit the migration and invasion properties of the M2-like TAMs (Figure 5a–f). Similar results were observed in the post-dosing group (Supplementary Figure S3a–f). The above results reveal that SAA inhibited the M2-like TAMs’ migration and invasion capacity induced by the TCM-TNBC by promoting M2-like TAM re-polarization towards M1-like TAMs.

### 2.6. SAA Inhibited TGF-β1 Expression and ERK Signalling in M2-like TAMs

**Figure 6:**
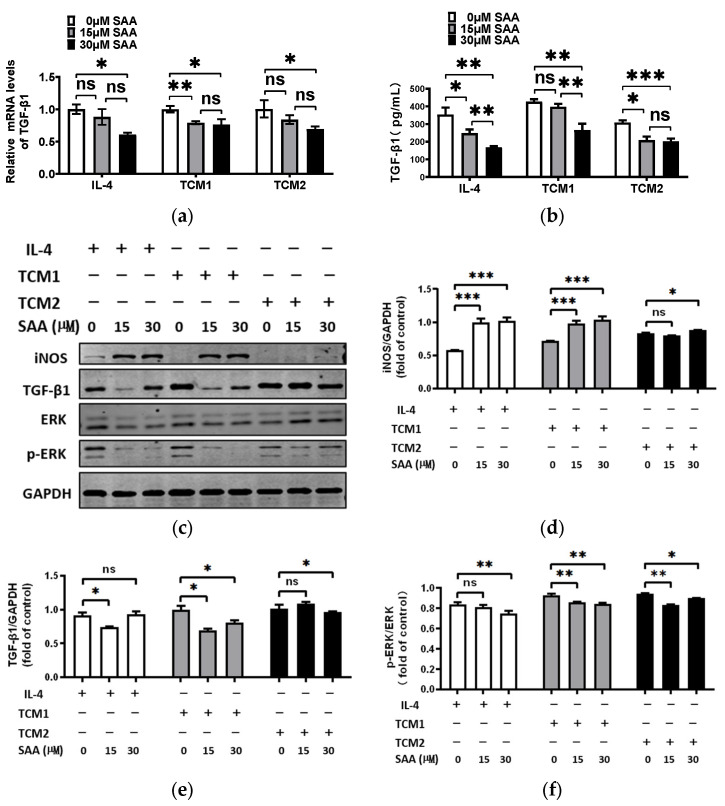
SAA inhibited TGF-β1 expression and ERK signaling in M2-like TAMs. (**a**) TGF-β1 mRNA levels were analyzed by qRT-PCR. (**b**) The secretion of TGF-β1 was measured by ELISA assay. (**c**–**f**) The expression levels of iNOS, TGF-β1, ERK, and p-ERK in M2-like TAMs were evaluated by Western blot. * *p* < 0.05; ** *p* < 0.01; *** *p* < 0.001.

In cancer, M2-like TAMs express TGF-β, which promotes tumor growth [22], and TGF-β can activate M2c TAMs [23,24], creating a cycle of continuous activation of M2 TAMs and promoting tumor progression. We investigated the expression level of TGF-β1 in M2-like TAMs induced by the TNBC-TCM treated with SAA. The results showed a decrease in the mRNA and protein expression levels of TGF-β1 after treatment with SAA (Figure 6a,b). Interestingly, our results showed that after treatment with SAA, the protein expression level of iNOS was increased and p-ERK and TGF-β1 were reduced, while the total ERK remained unaffected (Figure 6c). The results maintained a consistent trend in the post-dosing group (Supplementary Figure S4a–f). 

ERK signaling has been shown to play a key role in hypoxia-induced M2-like TAMs polarization [25] and activation of ERK signaling can drive the polarization of M2-like TAMs through various mechanisms in the TME [26]. Our results indicated that SAA inhibits TGF-β1 expression and ERK signaling in M2-like TAMs.

## 3. Discussion

Macrophages derived from peripheral blood monocytes transform into tumor-associated macrophages (TAMs) upon migration to the tumor site [27]. Under the influence of various microenvironmental factors, macrophages can be classified into two subtypes: M1 TAMs and M2 TAMs [28]. M1 TAMs, known as classically activated macrophages, are associated with the pro-inflammatory response and antimicrobial activity, producing high levels of iNOS and secreting pro-inflammatory cytokines, such as CD86 and IL-1β. M2 TAMs, known as alternatively activated macrophages, primarily participate in hypersensitivity reactions and antiparasitic immune responses, producing high levels of CD206 and Arg-1 and secreting anti-inflammatory cytokines, such as TGF-β1 [29]. It has been shown that as the tumor progresses, the TAMs in the tumor tissue eventually show a predominance of M2 TAMs [30]. M2 TAMs play a crucial role in the tumor microenvironment by mediating inflammation and facilitating tumor initiation and progression [31,32,33]. Based on the applied stimuli and the achieved transcriptional changes, the M2 TAMs can be classified into four subdivisions: M2a, M2b, M2c, and M2d [34,35]. The most commonly described M2 TAM is the alternatively activated macrophage (M2a), which can be generated by IL-4/IL-13. IL-4 greatly stimulates the expression of mannose receptors (MRC1, CD206), which is widely accepted as the canonical marker for M2a TAMs [15].

Notably, TAMs are not static but are a group of dynamically changing cells that exhibit remarkable plasticity, closely related to their cytokine environment, the degree of hypoxia within the tumor, tumor typing, staging, and treatment [36]. They can be converted from M1 TAMs to M2 TAMs or vice versa in response to changes in the local environment [37], for example, a high-fat diet can shift adipose tissue macrophages from the M2 TAMs to the M1 TAMs and exercise can reverse this process [38]. One potential approach to inhibit tumor progression is to reverse or inhibit the polarization of M2 TAMs that support pro-tumor progression [31]. The mouse macrophage cell line RAW264.7, the most commonly used mouse macrophage cell line in medical research, has been used extensively to study the functions, mechanisms, and signaling pathways of macrophages [39]. In our study, RAW264.7 was polarized into M2 TAMs with IL-4 for the positive model of M2 TAMs [40,41] and we chose to determine M1 TAMs by detecting CD86, iNOS and IL-1β [42], and M2 TAMs by detecting CD206 and Arg-1 [43].

We first induced RAW264.7 polarize to M2 TAMs by co-culturing them with triple-negative breast cancer cell conditioned media (TCM-TNBC: TCM-SUM159 and TCM-4T1) so as to mimic TAMs in the tumor microenvironment. We found that after TCM-TNBC inducing, the mRNA expression of M1 TAM markers (CD86, IL-1β, and iNOS) was significantly reduced, whereas the expression of M2 TAM markers (Arg-1) was significantly increased and the protein expression of iNOS was significantly reduced, whereas the expression of Arg-1 was significantly elevated, which suggests that TCM-TNBC successfully induced M2-like TAM polarization (Figure 1).

Using the Timber 2.0 database, we found a positive correlation between TGF-β1 expression and M2 macrophage infiltration in human breast cancer cells. TGF-β1 is a potent inducer of EMT [44], which promotes the invasion and metastasis of tumor cells by acting on both tumor cells and immune cells in the tumor microenvironment [14] and is associated with a poor prognosis in a variety of tumor types [45]. M2 TAMs can express high levels of TGF-β1, which can selectively activate M2 TAMs, thus creating a vicious cycle [14] that continuously stimulates the production of M2 TAMs. We observed increased protein expression levels of TGF-β1 and p-ERK in M2-like TAMs (Figure 2). The ERK signaling pathway has been reported to be involved in stimulating the polarization of M2-like TAMs as a driver of breast cancer progression [15].

Salvianolic acid A (SAA) and salvianolic acid B (SAB), as natural compounds from Chinese herbs, have emerged as novel anti-cancer paradigms for multi-targeted cancer prevention [9]. The anti-cancer effects of SAA and SAB were found to be associated with mediating the intrinsic apoptotic pathway and targeting the PI3K/Akt pathway [9]. SAB has been shown to have anti-proliferative properties against colorectal cancer [46], head and neck carcinoma [47], and hepatocellular cancer [48]. SAA effectively suppresses the proliferation of several breast cancer cell lines, such as MCF-7, MDA-MB-231, and MDA-MB-453 [49]. Studies have also shown that SAA reverses paclitaxel resistance in human breast cancer [50], reduces MMP-2 expression, and inhibits p-ERK protein expression in human nasopharyngeal carcinoma cells [51] and oral squamous cell carcinoma [52]. Besides its anti-cancer application, SAA has other biomedical applications, such as anti-inflammatory, antioxidant, neuroprotective, and antidepressant effects [53].

Many herbal components have been shown to play a role in TAM polarization, such as andrographolide [54] and curcumin [55]; however, not much is known regarding the effects of SAA on TAMs. In our study, we hypothesized that SAA could inhibit M2-like TAM polarization from a pro-tumor phenotype to an anti-tumor phenotype after treating macrophages with a TNBC-cell-conditional medium.

Using a series of experiments including qPCR, WB, ELISA, IF, and FCM, we found that SAA inhibited the M2-like polarization of macrophages by reducing the expression levels of Arg-1 and TGF-β1, as well as by upregulating the expression levels of CD86, IL-1β, and iNOS. iNOS production appears to be reduced after TAMs switch to M2-like TAMs, thus inhibiting the ability of M1 TAMs to destroy tumor cells [56]. Furthermore, SAA treatment of M2-like TAMs resulted in a concentration-dependent decrease in CD206. These results indicate that SAA may play a role in inhibiting the polarization of M2-like TAMs induced by TCM-TNBC. SAA subsequently suppresses the invasion and migration of M2-like TAMs. Combined with the fact that SAA suppressed the observed malignant behavior of M2-like TAMs, it was proposed that SAA may exert anti-tumor effects by inhibiting M2-like TAM polarization and promoting M1-like TAM polarization. In M2-like TAMs induced by TNBC-TCM, we observed that the mRNA expression level of TGF-β1 was significantly increased but decreased with the use of SAA in a concentration-dependent manner and the protein expression levels of TGF-β1 and p-ERK were also decreased. This suggests that the ERK pathway plays a role in the reprogramming of M2 TAMs by SAA.

Although the present study provides evidence for the mechanism of SAA reprogramming of M2-like TAMs, there are still some limitations that should be highlighted. Firstly, this study relied solely on in vitro experiments, so further validation of the in vivo effect of SAA is required in future studies. Secondly, although this study focused on the ERK pathway, it is crucial to explore whether other mechanisms contribute to the reprogramming of M2-like TAMs via SAA. In addition, it would be worthwhile to investigate the potential synergistic effects of SAA in combination with other drugs to further enhance its therapeutic efficacy. Finally, broadening the application areas of SAA by exploring its potential effects on other diseases warrants further investigation by researchers. 

**Figure 7:**
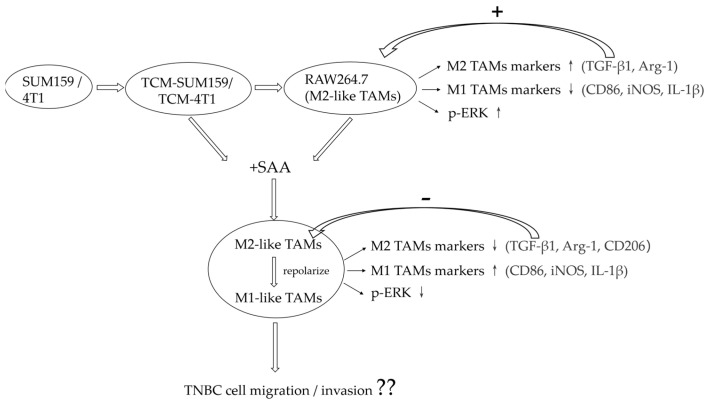
Experimental mechanisms.

In summary, the present study reported that SAA treatment promotes the expression of M1 TAM markers (CD86, iNOS, and IL-1β) and inhibits the expression of M2 TAM markers (Arg-1, CD206, and TGF-β1) and the protein level of p-ERK (Figure 7). The effects of different phenotypes of TAMs on TNBC migration or invasion will also be studied in the follow-up. This suggests that SAA may be able to reprogram M2-like TAMs towards M1-like TAMs by down-regulating the ERK pathway. This study may provide new insights into the therapeutic potential of SAA in the treatment of TNBC and other malignancies.

## 4. Materials and Methods

### 4.1. Chemicals and Reagents 

Salvianolic acid A (SAA, 96574-01-5) was purchased from Sigma-Aldrich, Shanghai, China. The SAA used in this study had a purity of ≥95.0%, as determined via HPLC analysis. SAA was dissolved in dimethyl sulfoxide (DMSO; Sigma-Aldrich, Shanghai, China) to prepare a stock solution. In order to mitigate potential solvent effects in all cell experiments, DMSO was included in the control group during in vitro studies. The final concentrations of DMSO in all culture wells were maintained below 0.05% (*v*/*v*).

IL-4 (HY-P7042) was purchased from MCE, Monmouth Junction, NJ, USA.

### 4.2. Cell Lines and Culture

The human TNBC line SUM159, mouse mammary gland tumor cells 4T1, and mouse macrophage RAW264.7 cells were generously provided by the Stem Cell Bank, the Chinese Academy of Sciences. These cells were cultured in DMEM (high glucose) supplemented with 10% fetal bovine serum (FBS) at 37 °C in a humidified incubator with 5% CO_2_. The experiment utilized logarithmic phase cells for the co-culture experiment.

### 4.3. Preparation of Tumor-Conditioned Medium (TCM)

TNBC cells (SUM159 or 4T1) were starved at 70% confluency for 24 h; then, the cell supernatants were collected and filtered through a 0.45 μm filter to remove cell debris and then stored at −20 °C for further use [55,57,58]. 

Before use, we additionally added 10% FBS after freezing at room temperature. 

**Figure 8:**
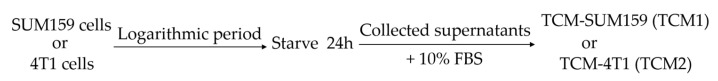
Preparation of tumor-conditioned medium (TCM).

We referred to TCM-SUM159 as TCM1 and TCM-4T1 as TCM2 (Figure 8).

### 4.4. Polarization of TAMs

IL-4 was employed to induce RAW264.7 polarization into M2 TAMs for the positive control group [15]. When the RAW264.7 cells reached the logarithmic phase of growth, macrophages were polarized into M2 TAMs using 20 ng/mL of IL-4 for 24 h. 

When the RAW264.7 cells reached the logarithmic phase of growth, macrophages were co-cultured with TCM-SUM159 (TCM1) and TCM-4T1 (TCM2) for 24 h and 48 h, respectively, to polarize them into M2-like TAMs.

**Figure 9:**
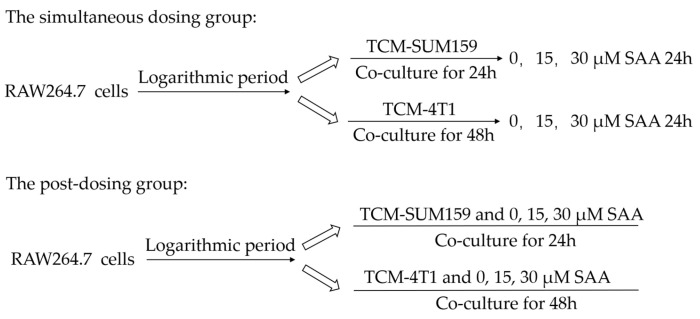
Experimental group design.

For the simultaneous dosing group, RAW264.7 cells were polarized into M2 TAMs by IL-4, TCM1, or TCM2 in the presence of a vehicle or various concentrations of SAA (Figure 9). 

For the post-dosing group, after polarization of macrophages to M2 TAMs using IL-4, TCM1, or TCM2, the medium was discarded and cells were washed twice with PBS. Then, different concentrations of SAA were added to the culture medium and incubated for 24 h (Figure 9).

### 4.5. Cell Viability Assay

The half-inhibitory concentration (IC50) for SAA in RAW264.7 cells was assessed using Cell Counting Kit 8 (CCK-8; CK04, DOJINDO, Kumamoto, Japan). Firstly, RAW264.7 cells were seeded at a density of 8 × 10^3^ cells/well in 96-well plates during the logarithmic phase, followed by overnight incubation. Cells were then treated with different concentrations (0, 15, 30, 60, 100, 200, 400, and 1000 μM) of SAA for determination of IC50, while cells were treated with IL-4 (20 ng/mL, 24 h), TCM-SUM159 (TCM1, 24 h), and TCM-4T1 (TCM2, 48 h) in the presence of the vehicle or SAA (0, 15, 30, 45, and 60 μM) for the simultaneous dosing groups. For the post-dosing group, cells were treated with IL-4 (20 ng/mL, 24 h), TCM-SUM159 (TCM1, 24 h), and TCM-4T1 (TCM2, 48 h) followed by SAA (0, 15, 30, 45, and 60 μM). After 24 h treatment with SAA, a total of 10 μL of Cell Counting Kit-8 reagent was then added to each well to evaluate cell viability. Subsequently, the absorbance values at 450 nm were measured using a microplate reader (Molecular Devices, San Jose, CA, USA).

### 4.6. RNA Extraction and qRT-PCR

Total RNA was extracted from the cells using a Trizol reagent (15596026CN; Ambion, Austin, TX, USA) based on the manufacturer’s instructions. The quality was assessed via a Thermo NanoDrop8000. Then, the RNA was reverse-transcribed into cDNA via a PrimeScriptTM RT Reagent Kit (TaKaRa Bio, Dalian, China). A TB Green Premix Ex Taq II kit (TaKaRa Bio, Dalian, China) was used to carry out quantitative real-time PCR (qRT-PCR) with an Applied Biosystems 7500 Real-Time PCR System (Applied Biosystems, Singapore).

Each sample was individually run in triplicate. The primer sequences and corresponding products are listed in Table 1.

The amplification conditions were as follows: 95 °C for 30 s followed by 40 cycles consisting of 95 °C for 5 s, 60 °C for 34 s, 95 °C for 15 s, 60 °C for 1 min, and 95 °C for 15 s. Ct values were analyzed using the 2^−ΔΔCt^ method and normalized to the mean Ct value of β-actin.

### 4.7. Enzyme-Linked Immunosorbent Assay (ELISA)

Enzyme-linked immunosorbent assays (ELISA) were used to detect the concentrations of cell-secreted TGF-β1 (CSB-E04741m, BOSTER, Wuhan, China). The ELISA assays were performed according to the manufacturer’s instructions.

### 4.8. Immunofluorescence Staining

A cell climbing slice was prepared and placed into a 24-well plate. When cells were attached to the wall, they were divided into two groups: the control group and two SAA groups. After incubating for the corresponding time, the cells were then washed three times for 5 min each with cold PBS buffer and fixed with 4% formaldehyde at room temperature for 30 min. Next, the cells were permeabilized with 0.2% Triton-X 100 in PBS for 10 min at room temperature. The serum was diluted to 5% with PBS and incubated with the cells at room temperature for 1 h. Following this, all samples were incubated with the primary antibodies iNOS (dilution,1:200; Proteintech, Wuhan, China) and CD206 (dilution, 1:200; Proteintech, Wuhan, China) at 4 °C overnight. After gently rinsing the cells three times with PBS, they were incubated at 37 °C for 1.5 h with Mouse IgG(H + L)/FITC (dilution, 1:50, ZN-0312; ZSGB-BIO, Beijing, China) and Rabbit IgG(H + L)/FITC (dilution, 1:50, ZN-0311; ZSGB-BIO) as secondary antibodies. Additionally, the nuclei were counterstained with 5 μg/mL of diaminophenylindole (DAPI; Sigma-Aldrich, Shanghai, China) for 5 min. Washing steps were performed three times for 5 min after each step. Finally, immunofluorescence staining was visualized using a fluorescence microscope (Leica DMi8, Wetzlar, Germany).

### 4.9. Flow Cytometry

The proportion of CD86+ and CD206+ M2 macrophages was detected by flow cytometry. The cells were washed with PBS containing 1% FBS (wash buffer) and then resuspended at a density of 10^6^ cells/100 mL of PBS containing 1% FBS. The suspensions were blocked with 5 μL of CD16/32 (101319, Biolegend, San Diego, CA, USA) at 4 °C for 15 min. To evaluate the M2 TAMs, the cells were labeled with CD86 (105007, Biolegend, San Diego, CA, USA) in the dark at 4 °C for 30 min. After centrifugation at 1000 rpm for 5 min, the supernatants were removed and fixation buffer (420801, Biolegend, San Diego, CA, USA) was added in the dark at room temperature for 20 min. Fixed cells were resuspended in diluted intracellular staining permeabilization wash buffer (1×) and centrifuged at 300× *g* for 10 min, repeating the process twice. The suspensions were incubated with CD206 (141707, Biolegend, San Diego, CA, USA) in the dark at 4 °C for 30 min, followed by a final resuspension in 200 μL of PBS for the assay. Fluorescence-activated cell sorting (FACS) was used to measure the stained cells. Flow cytometry was performed using a NovoCyte flow cytometer (ACEA NovoCyteTM2070R, Singapore).

### 4.10. Transwell Assay

RAW264.7 cells were suspended in 100 μL of TCM1, TCM2, or IL-4 at a cell density of 1 × 10^6^/mL in the presence of the vehicle or SAA (15 and 30 μM) and then added to the upper chambers (8 μm; Corning, NY, USA) of a 24-well Transwell plate for 24 h. A volume of 600 μL of DMEM containing 20% FBS was added to the lower chamber. After incubation for 24 h at 37 °C and 5% CO_2_, the upper chamber was fixed in methanol for 20 min at room temperature and air-dried, followed by staining with 0.1% crystal violet solution for 20 min. The number of cells in five randomly selected fields (200×) was counted under a microscope (Leica DMi8, Wetzlar, Germany) and statistically analyzed. For the Transwell invasion assay, Matrigel (Corning, NY, USA) was applied to precoat the upper chamber of the Transwell plates for 2 h. The remaining steps were the same as the migration experiment.

### 4.11. Western Blot

The cells were cultured with IL-4, TCM1, or TCM2 for the corresponding time to polarize into M2-like TAMs in the presence of the vehicle or SAA (15 and 30 μM). Subsequently, the cells were harvested and lysed with RIPA lysis buffer (P0013B, Beyotime, Shanghai, China) containing PMSF (10837091001, Roche, Shanghai, China). Protein concentrations were determined using a BCA protein assay kit (P0009, Beyotime). Proteins (40 μg) were separated via 10% SDS-PAGE and transferred to nitrocellulose filter (NC) membranes (0.45 μM, 71151558; Pall Corporation, Temixco, Mexico). The membranes were then blocked with 5% BSA-TBST for 1 h and subsequently incubated overnight at 4 °C with the primary antibodies GAPDH (1:20,000 dilution, 60004-1-Ig; Proteintech, San Diego, CA, USA), iNOS (1:1000 dilution 18985-1-AP; Proteintech), Arg-1 (1:1000 dilution, 66129-1-Ig; Proteintech), TGF-β1 (1:1000 dilution, 21898-1-AP; Proteintech), ERK (1:500 dilution, 4695S; CST, Shanghai, China), and p-ERK (1:250 dilution, 4370S; CST). GAPDH was used as the internal reference. The bands were washed three times with TBST (0.1% Tween-20) for 10 min each at room temperature and treated with IRDye^®^ 800CW Donkey anti-Mouse IgG (1:10,000; LI-COR, Lincoln, NE, USA) or IRDye^®^ 800CW Donkey anti-Rabbit IgG (1:10,000; LI-COR). Finally, the protein bands were visualized using an Odyssey CLX-0800 (LI-COR, USA) and analyzed using Image J software (Version 1.54i 03).

### 4.12. Bioinformatics Analysis

The TIMER 2.0 database (https://cistrome.shinyapps.io/timer/) TIMER2.0 (http://timer.comp-genomics.org/timer/) (accessed on 9 December 2023) was used to analyze the correlation between TGF-β1 and tumor-infiltrating M2 macrophages in human breast cancer.

### 4.13. Statistical Analysis

All data are presented as the mean ± standard deviation of at least triplicate experiments and analyzed utilizing Prism 9.0 software (GraphPad Software, Boston, MA, USA). Significant differences between groups were calculated using Student’s *t*-tests or analysis of variance (ANOVA). A *p*-value < 0.05 was considered statistically significant.

## Figures and Tables

**Table 1:** Primers used for qRT-PCR.

Gene	Primer (5′-3′)
β-actin	F: TGC CGC ATC CTC TTC CTC R: GCC ACA GGA TTC CAT ACC C
CD86	F: ATG GGC TCG TAT GAT TGT TT
	R: CGG GTG ACC TTG CTT AGA C
IL-1β	F: CAA CCA ACA AGT GAT ATT CTC CAT G
	R: GAT CCA CAC TCT CCA GCT GCA
iNOS	F: GAA ACG CTT CAC TTC CAA TG
	R: GTG GTG CGG CTG GAC TTT
IL-6	F: CTT GGG ACT GAT GCT GGT G
	R: TTG CCA TTGCAC AAC TCT TT
Arg-1	F: TGC TCA CAC TGA CAT CAA CAC T
	R: CTA CGT CTC GCA AGC CAA T
TGF-β1	F: GCG GAC TAC TAT GCT AAA GAG G
	R: CAC TGC TTC CCG AAT GTC T

## Data Availability

Data are contained within the article and Supplementary Materials. The datasets used and analyzed during this study are available from the corresponding author upon reasonable request (ywl0621@swmu.edu.cn).

## Supplementary Materials

The following supporting information can be downloaded at: https://www.mdpi.com/article/10.3390/molecules29071469/s1. Figure S1: SAA mediated the mRNA expression levels of cytokines in M2-like TAMs induced by the TNBC-cell-conditioned medium. (a) We named TCM-SUM159 as TCM1; TCM-4T1 as TCM2. RAW264.7 cells were first exposed to IL-4 (24 h), TCM1 (24 h) and TCM2 (48 h), then treated with SAA (0–60 μM) for 24 h, the cell viability of the M2-like TAMs were detected by CCK-8. ** *p* < 0.01, *** *p* < 0.001, **** *p* < 0.0001 versus control. (b) RAW264.7 were treated with SAA, the relative mRNA expression of CD86, IL-1β and iNOS were measured by qRT-PCR. * *p* < 0.05; ** *p* < 0.01; *** *p* < 0.001; **** *p* < 0.0001. (c–f) Afte IL-4, TCM1 and TCM2 induction, RAW264.7 were then treated with SAA, the relative mRNA expression of CD86, IL-1β, iNOS and Arg-1 were measured by qRT-PCR. * *p* < 0.05; ** *p* < 0.01; *** *p* < 0.001; **** *p* < 0.0001. IL-4 group was used as a positive control. M1 TAMs markers: CD86, IL-1β, iNOS; M2 TAMs markers: Arg-1; Figure S2: SAA inhibited the progression of RAW264.7 cell polarization towards M2-like TAMs. (a,b) Immunofluorescence staining was performed to detect expression of iNOS and CD206, which is shown in green, while the nuclei appear in blue (×400). (c,d) Flow cytometric analysis of surface markers of CD86 and CD206 in M2-like TAMs. * *p* < 0.05; ** *p* < 0.01; *** *p* < 0.001; **** *p* < 0.0001. IL-4 was used as a positive control. M1 TAMs markers: CD86, iNOS; M2 TAMs markers: CD206; Figure S3: SAA inhibited the migration and invasion of M2-like TAMs induced by the TCM-TNBC. IL-4(a-b), TCM1(c-d), or TCM2(e-f), induced M2-like TAMs while adding 15 or 30 μ M SAA for migration and invasion assays. * *p* < 0.05; ** *p* < 0.01; *** *p* < 0.001; **** *p* < 0.0001; Figure S4: SAA inhibited TGF-β1 expression and ERK signaling in M2-like TAMs. (a) TGF-β1 mRNA levels were analysed by qRT-PCR. (b) The secretion of TGF-β1 was measured by ELISA. (c,d) The expression of iNOS, TGF-β1, ERK and p-ERK in M2-like TAMs were evaluated through Western Blot. * *p* < 0.05; ** *p* < 0.01; *** *p* < 0.001; **** *p* < 0.0001.

## Abbreviations

TME	tumor microenvironment
TAM	tumor-associated macrophage
TNBC-TCM	triple-negative-breast-cancer-cell-conditioned medium
SAA	salvianolic acid A
TCM1	SUM159-conditioned medium
TCM2	4T1-conditioned medium

## References

[1] Pedersen R.N., Esen B., Mellemkjær L., Christiansen P., Ejlertsen B., Lash T.L., Nørgaard M., Cronin-Fenton D. (2022). The Incidence of Breast Cancer Recurrence 10-32 Years After Primary Diagnosis. JNCI J. Natl. Cancer Inst..

[2] Siegel R.L., Giaquinto A.N., Jemal A. (2024). Cancer statistics, 2024. CA Cancer J. Clin..

[3] Thakur K.K., Bordoloi D., Kunnumakkara A.B. (2018). Alarming Burden of Triple-Negative Breast Cancer in India. Clin. Breast Cancer.

[4] Kim J., Bae J.-S. (2016). Tumor-Associated Macrophages and Neutrophils in Tumor Microenvironment. Mediat. Inflamm..

[5] Pittet M.J., Michielin O., Migliorini D. (2022). Clinical relevance of tumour-associated macrophages. Nat. Rev. Clin. Oncol..

[6] Ojalvo L.S., Whittaker C.A., Condeelis J.S., Pollard J.W. (2010). Gene expression analysis of macrophages that facilitate tumor invasion supports a role for Wnt-signaling in mediating their activity in primary mammary tumors. J. Immunol..

[7] Kowal J., Kornete M., A Joyce J. (2019). Re-education of macrophages as a therapeutic strategy in cancer. Immunotherapy.

[8] Lian-Niang L., Rui T., Wei-Ming C. (1984). Salvianolic Acid A, a New Depside from Roots of Salvia miltiorrhiza. Planta Med..

[9] Qin T., Rasul A., Sarfraz A., Sarfraz I., Hussain G., Anwar H., Riaz A., Liu S., Wei W., Li J. (2019). Salvianolic acid A & B: Potential cytotoxic polyphenols in battle against cancer via targeting multiple signaling pathways. Int. J. Biol. Sci..

[10] Martinez F.O., Helming L., Gordon S. (2009). Alternative activation of macrophages: An immunologic functional perspective. Annu. Rev. Immunol..

[11] Zhang J., Liu X., Wan C., Liu Y., Wang Y., Meng C., Zhang Y., Jiang C. (2020). NLRP3 inflammasome mediates M1 macrophage polarization and IL-1β production in inflammatory root resorption. J. Clin. Periodontol..

[12] Wisitpongpun P., Potup P., Usuwanthim K. (2022). Oleamide-Mediated Polarization of M1 Macrophages and IL-1β Production by Regulating NLRP3-Inflammasome Activation in Primary Human Monocyte-Derived Macrophages. Front. Immunol..

[13] Sica A., Bronte V. (2007). Altered macrophage differentiation and immune dysfunction in tumor development. J. Clin. Investig..

[14] Fuxe J., Karlsson M.C. (2012). TGF-β-induced epithelial-mesenchymal transition: A link between cancer and inflammation. Semin. Cancer Biol..

[15] Huang X., Li Y., Fu M., Xin H.B. (2018). Polarizing Macrophages In Vitro. Methods Mol. Biol..

[16] Dong P., Ma L., Liu L., Zhao G., Zhang S., Dong L., Xue R., Chen S. (2016). CD86^+^/CD206^+^, Diametrically Polarized Tumor-Associated Macrophages, Predict Hepatocellular Carcinoma Patient Prognosis. Int. J. Mol. Sci..

[17] Sun D., Luo T., Dong P., Zhang N., Chen J., Zhang S., Liu L., Dong L., Zhang S. (2020). CD86(+)/CD206(+) tumor-associated macrophages predict prognosis of patients with intrahepatic cholangiocarcinoma. PeerJ.

[18] Kinouchi M., Miura K., Mizoi T., Ishida K., Fujibuchi W., Ando T., Yazaki N., Saito K., Shiiba K.-I., Sasaki I. (2011). Infiltration of CD14-positive macrophages at the invasive front indicates a favorable prognosis in colorectal cancer patients with lymph node metastasis. Hepatogastroenterology.

[19] Beider K., Bitner H., Leiba M., Gutwein O., Koren-Michowitz M., Ostrovsky O., Abraham M., Wald H., Galun E., Peled A. (2014). Multiple myeloma cells recruit tumor-supportive macrophages through the CXCR4/CXCL12 axis and promote their polarization toward the M2 phenotype. Oncotarget.

[20] Zhang H., Wang X., Shen Z., Xu J., Qin J., Sun Y. (2015). Infiltration of diametrically polarized macrophages predicts overall survival of patients with gastric cancer after surgical resection. Gastric Cancer.

[21] Hu W., Qian Y., Yu F., Liu W., Wu Y., Fang X., Hao W. (2015). Alternatively activated macrophages are associated with metastasis and poor prognosis in prostate adenocarcinoma. Oncol. Lett..

[22] van Dalen F.J., Van Stevendaal M.H., Fennemann F.L., Verdoes M., Ilina O. (2018). Molecular Repolarisation of Tumour-Associated Macrophages. Molecules.

[23] Gordon S. (2003). Alternative activation of macrophages. Nat. Rev. Immunol..

[24] Martinez F.O., Sica A., Mantovani A., Locati M. (2008). Macrophage activation and polarization. Front. Biosci..

[25] Zhang J., Cao J., Ma S., Dong R., Meng W., Ying M., Weng Q., Chen Z., Ma J., Fang Q. (2014). Tumor hypoxia enhances Non-Small Cell Lung Cancer metastasis by selectively promoting macrophage M2 polarization through the activation of ERK signaling. Oncotarget.

[26] Mu X., Shi W., Xu Y., Xu C., Zhao T., Geng B., Yang J., Pan J., Hu S., Zhang C. (2018). Tumor-derived lactate induces M2 macrophage polarization via the activation of the ERK/STAT3 signaling pathway in breast cancer. Cell Cycle.

[27] Garg K., Pullen N.A., Oskeritzian C.A., Ryan J.J., Bowlin G.L. (2013). Macrophage functional polarization (M1/M2) in response to varying fiber and pore dimensions of electrospun scaffolds. Biomaterials.

[28] Locati M., Curtale G., Mantovani A. (2020). Diversity, Mechanisms, and Significance of Macrophage Plasticity. Annu. Rev. Pathol. Mech. Dis..

[29] Ohno S., Inagawa H., Dhar D.K., Fujii T., Ueda S., Tachibana M., Suzuki N., Inoue M., Soma G.-I., Nagasue N. (2003). The degree of macrophage infiltration into the cancer cell nest is a significant predictor of survival in gastric cancer patients. Anticancer Res..

[30] Ruffell B., Affara N.I., Coussens L.M. (2012). Differential macrophage programming in the tumor microenvironment. Trends Immunol..

[31] DeNardo D.G., Ruffell B. (2019). Macrophages as regulators of tumour immunity and immunotherapy. Nat. Rev. Immunol..

[32] Zhao C., Pang X., Yang Z., Wang S., Deng H., Chen X. (2022). Nanomaterials targeting tumor associated macrophages for cancer immunotherapy. J. Control. Release.

[33] Pan Y., Yu Y., Wang X., Zhang T. (2020). Tumor-Associated Macrophages in Tumor Immunity. Front. Immunol..

[34] Murray P.J., Allen J.E., Biswas S.K., Fisher E.A., Gilroy D.W., Goerdt S., Gordon S., Hamilton J.A., Ivashkiv L.B., Lawrence T. (2014). Macrophage activation and polarization: Nomenclature and experimental guidelines. Immunity.

[35] Mantovani A., Sica A., Sozzani S., Allavena P., Vecchi A., Locati M. (2004). The chemokine system in diverse forms of macrophage activation and polarization. Trends Immunol..

[36] Russell J.S., Brown J.M. (2013). The irradiated tumor microenvironment: Role of tumor-associated macrophages in vascular recovery. Front. Physiol..

[37] Yuan R., Li S., Geng H., Wang X., Guan Q., Li X., Ren C., Yuan X. (2017). Reversing the polarization of tumor-associated macrophages inhibits tumor metastasis. Int. Immunopharmacol..

[38] Kawanishi N., Yano H., Yokogawa Y., Suzuki K. (2010). Exercise training inhibits inflammation in adipose tissue via both suppression of macrophage infiltration and acceleration of phenotypic switching from M1 to M2 macrophages in high-fat-diet-induced obese mice. Exerc. Immunol. Rev..

[39] Hartley J.W., Evans L.H., Green K.Y., Naghashfar Z., Macias A.R., Zerfas P.M., Ward J.M. (2008). Expression of infectious murine leukemia viruses by RAW264.7 cells, a potential complication for studies with a widely used mouse macrophage cell line. Retrovirology.

[40] Mosser D.M., Zhang X. (2008). Activation of murine macrophages. Curr. Protoc. Immunol..

[41] Junttila I.S., Mizukami K., Dickensheets H., Meier-Schellersheim M., Yamane H., Donnelly R.P., Paul W.E. (2008). Tuning sensitivity to IL-4 and IL-13: Differential expression of IL-4Ralpha, IL-13Ralpha1, and gammac regulates relative cytokine sensitivity. J. Exp. Med..

[42] Wang W., Yuan H.Y., Liu G.M., Ni W.H., Wang F., Tai G.X. (2015). *Escherichia coli* Maltose-Binding Protein Induces M1 Polarity of RAW264.7 Macrophage Cells via a TLR2- and TLR4-Dependent Manner. Int. J. Mol. Sci..

[43] Wang S., Cao M., Xu S., Shi J., Mao X., Yao X., Liu C. (2019). Luteolin Alters Macrophage Polarization to Inhibit Inflammation. Inflammation.

[44] Dumitriu I.E., Dunbar D.R., Howie S.E., Sethi T., Gregory C.D. (2009). Human dendritic cells produce TGF-beta 1 under the influence of lung carcinoma cells and prime the differentiation of CD4+CD25+Foxp3+ regulatory T cells. J. Immunol..

[45] Mariathasan S., Turley S.J., Nickles D., Castiglioni A., Yuen K., Wang Y., Kadel E.E., Koeppen H., Astarita J.L., Cubas R. (2018). TGFβ attenuates tumour response to PD-L1 blockade by contributing to exclusion of T cells. Nature.

[46] Jing Z., Fei W., Zhou J., Zhang L., Chen L., Zhang X., Liang X., Xie J., Fang Y., Sui X. (2016). Salvianolic acid B, a novel autophagy inducer, exerts antitumor activity as a single agent in colorectal cancer cells. Oncotarget.

[47] Zhao Y., Guo Y., Gu X. (2011). Salvianolic Acid B, a potential chemopreventive agent, for head and neck squamous cell cancer. J. Oncol..

[48] Kan S., Cheung W.M., Zhou Y., Ho W.S. (2014). Enhancement of Doxorubicin Cytotoxicity by Tanshinone IIA in HepG2 Human Hepatoma Cells. Planta Medica.

[49] Chen X., Guo J., Bao J., Lu J., Wang Y. (2013). The anticancer properties of Salvia miltiorrhiza Bunge (Danshen): A systematic review. Med. Res. Rev..

[50] Zheng X., Chen S., Yang Q., Cai J., Zhang W., You H., Xing J., Dong Y. (2015). Salvianolic acid A reverses the paclitaxel resistance and inhibits the migration and invasion abilities of human breast cancer cells by inactivating transgelin 2. Cancer Biol. Ther..

[51] Chuang C.-Y., Ho Y.-C., Lin C.-W., Yang W.-E., Yu Y.-L., Tsai M.-C., Yang S.-F., Su S.-C. (2020). Salvianolic acid A suppresses MMP-2 expression and restrains cancer cell invasion through ERK signaling in human nasopharyngeal carcinoma. J. Ethnopharmacol..

[52] Fang C., Wu C., Chen P., Chang Y., Chuang C., Lai C., Yang S., Tsai L. (2018). Antimetastatic potentials of salvianolic acid A on oral squamous cell carcinoma by targeting MMP-2 and the c-Raf/MEK/ERK pathway. Environ. Toxicol..

[53] Zhang H., Liu Y.Y., Jiang Q., Li K.R., Zhao Y.X., Cao C., Yao J. (2014). Salvianolic acid A protects RPE cells against oxidative stress through activation of Nrf2/HO-1 signaling. Free Radic. Biol. Med..

[54] Li L., Yang L., Yang S., Wang R., Gao H., Lin Z., Zhao Y., Tang W., Han R., Wang W. (2022). Andrographolide suppresses breast cancer progression by modulating tumor-associated macrophage polarization through the Wnt/*β*-catenin pathway. Phytother. Res..

[55] Jiang M., Qi Y., Huang W., Lin Y., Li B. (2022). Curcumin Reprograms TAMs from a Protumor Phenotype towards an Antitumor Phenotype via Inhibiting MAO-A/STAT6 Pathway. Cells.

[56] Weigert A., Brüne B. (2008). Nitric oxide, apoptosis and macrophage polarization during tumor progression. Nitric Oxide.

[57] Deswal B., Bagchi U., Kapoor S. (2024). Curcumin Suppresses M2 Macrophage-derived Paclitaxel Chemoresistance through Inhibition of PI3K-AKT/STAT3 Signaling. Anti-Cancer Agents Med. Chem..

[58] Ge S., Sun X., Sang L., Zhang M., Yan X., Ju Q., Ma X., Xu M. (2023). Curcumin inhibits malignant behavior of colorectal cancer cells by regulating M2 polarization of tumor-associated macrophages and metastasis associated in colon cancer 1 (MACC1) expression. Chem. Biol. Drug Des..

