# Supplementary material for: The Effect of Salvianolic Acid A on Tumor-Associated Macrophage Polarization and Its Mechanisms in the Tumor Microenvironment of Triple-Negative Breast Cancer
Source: Molecules. 2024 Mar 26;29(7):1469. doi: 10.3390/molecules29071469 (PMC11013304; doi:10.3390/molecules29071469)
Supplement: Supplementary file 1 [file molecules-29-01469-s001.zip › molecules-2813752-supplementary.pdf]

Supplementary Figures S1-S4

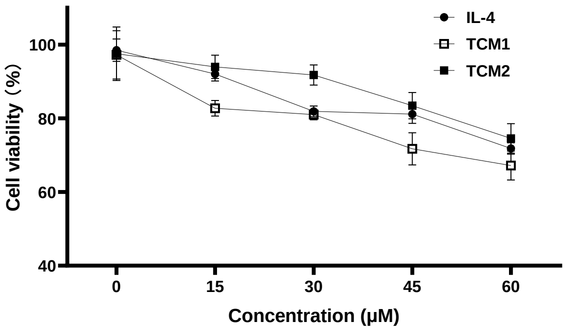

(a)

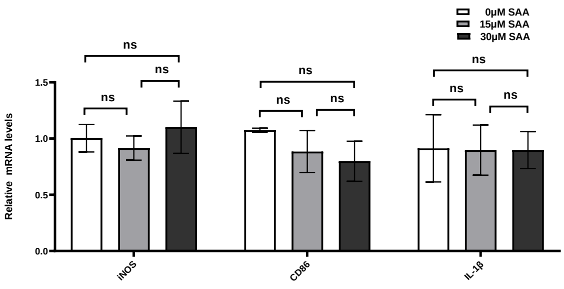

(b)

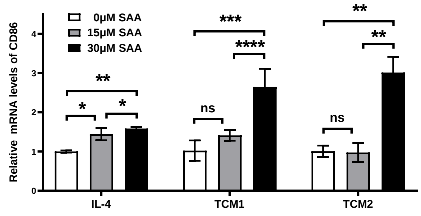

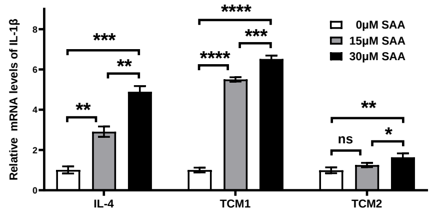

(c) **(d)**

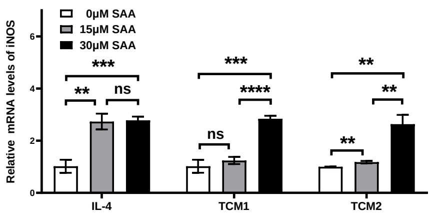

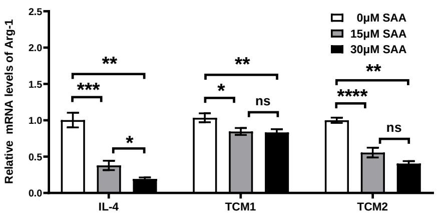

(e) **(f)**

Figure S1. SAA mediated the mRNA expression levels of cytokines in M2-like TAMs induced by the TNBC-cell-conditioned medium. (a) We named TCM-SUM159 as TCM1; TCM-4T1 as TCM2. RAW264.7 cells were first exposed to IL-4 (24h) , TCM1 (24h) and TCM2 (48h), then treated with SAA (0–60μM) for 24h, the cell viability of the M2-like TAMs were detected by CCK-8. **p<0.01, ***p<0.001, **** p < 0.0001 versus control. (b) RAW264.7 were treated with SAA, the relative mRNA expression of CD86, IL-1β and iNOS were measured by qRT-PCR. *p < 0.05; ** p < 0.01; *** p < 0.001; **** p < 0.0001. (c-f) After

IL-4, TCM1 and TCM2 induction, RAW264.7 were then treated with SAA, the relative mRNA expression of CD86, IL-1β, iNOS and Arg-1 were measured by qRT-PCR. *$p < 0.05$; ** $p < 0.01$; *** $p < 0.001$; **** $p < 0.0001$. IL-4 group was used as a positive control. M1 TAMs markers: CD86, IL-1β, iNOS; M2 TAMs markers: Arg-1.

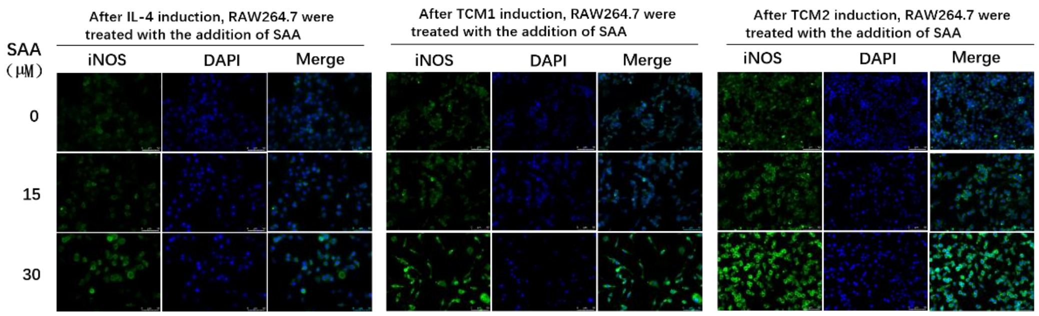

(a)

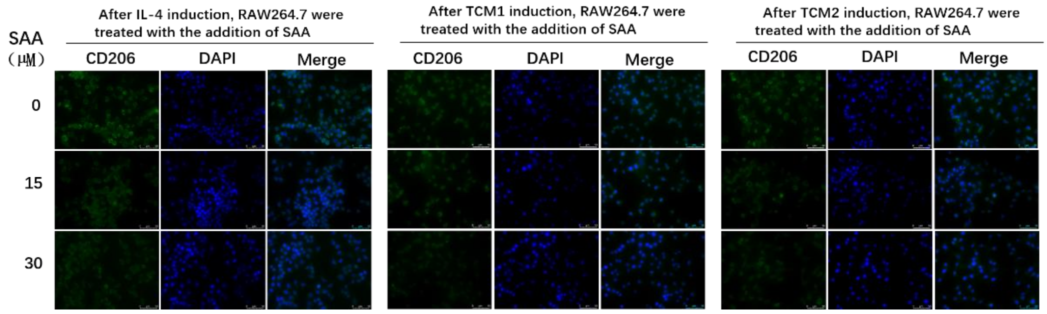

(b)

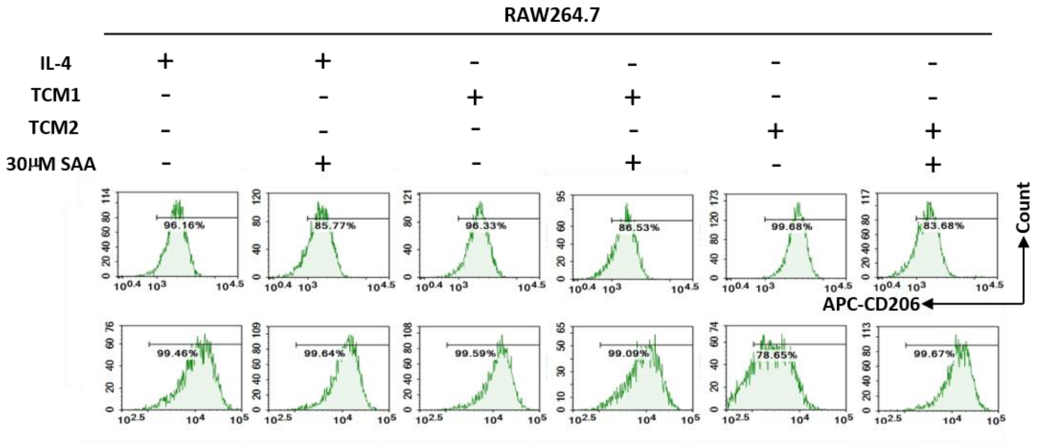

(c)

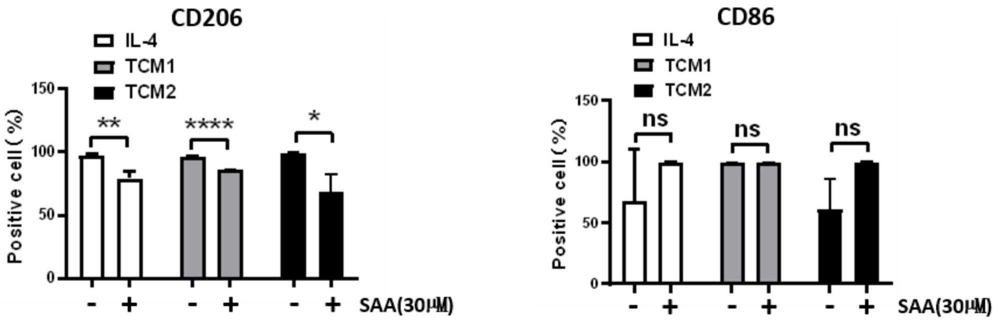

(d)

Figure S2. SAA inhibited the progression of RAW264.7 cell polarization towards M2-like TAMs. (a-b) Immunofluorescence staining was performed to detect expression of iNOS and CD206, which is shown in green, while the nuclei appear in blue (×400). (c-d) Flow cytometric analysis of surface markers of CD86 and CD206 in M2-like TAMs. * $p < 0.05$; ** $p < 0.01$; *** $p < 0.001$; **** $p < 0.0001$. IL-4 was used as a positive control. M1 TAMs markers: CD86, iNOS; M2 TAMs markers: CD206.

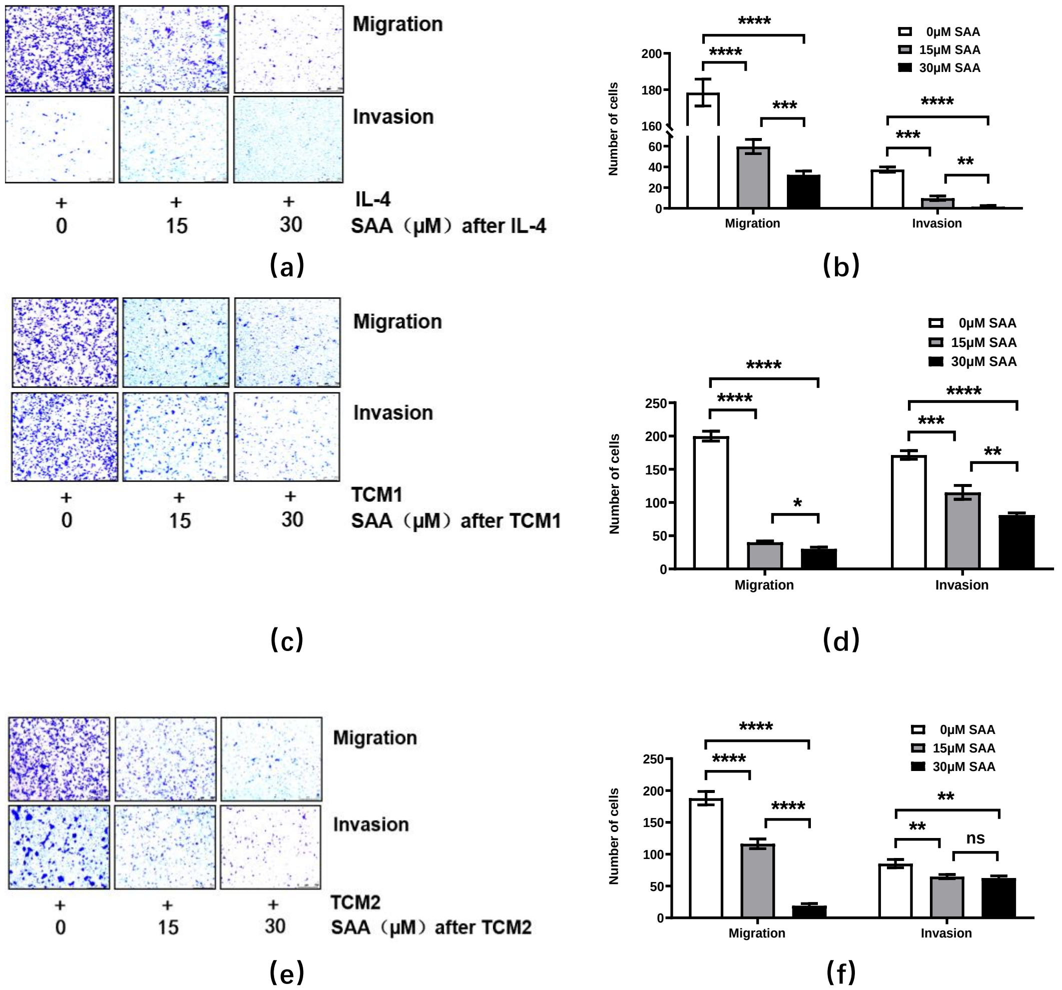

Figure S3. SAA inhibited the migration and invasion of M2-like TAMs induced by the TCM-TNBC. IL-4(a-b), TCM1(c-d), or TCM2(e-f), induced M2-like TAMs while adding 15 or 30 μM SAA for migration and invasion assays. * $p < 0.05$; ** $p < 0.01$; *** $p < 0.001$; **** $p < 0.0001$.

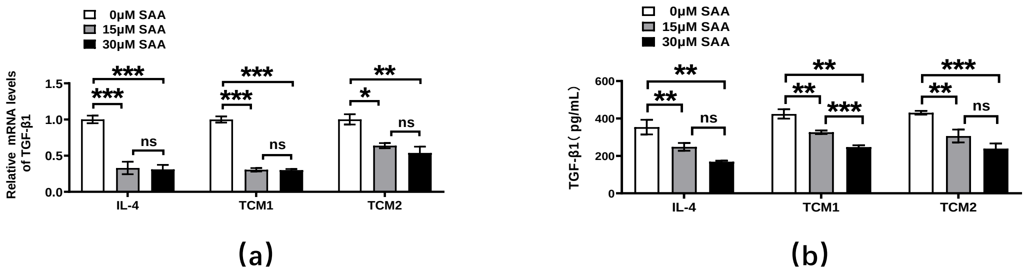

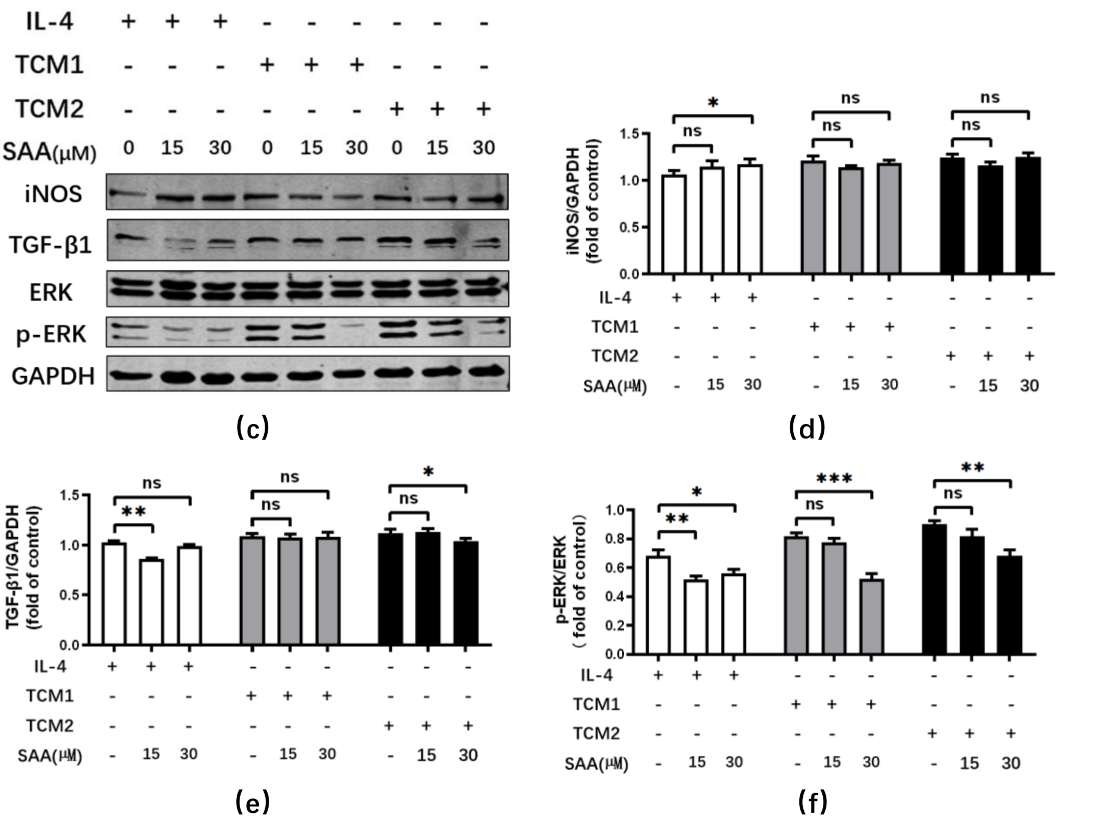

Figure S4. SAA inhibited TGF-β1 expression and ERK signaling in M2-like TAMs. (a) TGFβ1 mRNA levels were analysed by qRT-PCR. (b) The secretion of TGF-β1 was measured by ELISA. (c-d) The expression of iNOS, TGF-β1, ERK and p-ERK in M2-like TAMs were evaluated through Western Blot. * $p < 0.05$; ** $p < 0.01$; *** $p < 0.001$; **** $p < 0.0001$.
